# Structural and Functional Mise en Abyme

**DOI:** 10.3389/fmolb.2019.00131

**Published:** 2019-11-28

**Authors:** Bérangère Avalle, Séverine Padiolleau-Lefevre, Alain Friboulet

**Affiliations:** ^1^CNRS UMR 7025, Génie Enzymatique et Cellulaire. Centre de Recherche de Royallieu, Compiègne, France; ^2^Sorbonne Universités, Université de Technologie de Compiègne, Génie Enzymatique et Cellulaire. Centre de Recherche de Royallieu, Compiègne, France

**Keywords:** mimicry, abzyme, idiotypic network, autoimmunity, structure and function transfer

## Introduction

The idiotypic network of the immune system is known to have a regulatory role. A consequence of such a network lies on the internal image of the original antigen that anti-idiotypic antibodies represent. This characteristic has been exploited to generate anti-idiotypic catalytic antibodies (abzymes). Interestingly, the feasibility of such experiment has been initially demonstrated thanks to deliberate *in vitro* manipulations of the immune system and subsequently hypothesized through *in vivo* observations. The successive rational selections of proteins or peptides show that the transfer of a structural information from a partner to another one is effective. To date, the mimicry properties of proteins and peptides are extensively studied and are increasingly being exploited, justifying their place for diagnostic, or therapeutic purposes. We herein put into perspective the concept of molecular mimicry based on the transfer of structural and functional information between mimics, and discuss some biological consequences.

## Proteins as structural and functional mimics

A good evidence for the mimicry properties of proteins is given by the induction of antibodies bearing enzymatic features. Since the introduction of the idiotypic network theory leading to spontaneous antibodies having an immune regulation aim (Jerne, 1974), it is understood that some anti-idiotypic antibodies may display structural features which may be the internal image of the original antigen. These features are largely investigated for the identification of cell surface receptors for hormones, neuropeptides or growth factors, and for the conception of new vaccines (Lopez-Requena et al., 2012; Segatori et al., 2018).

Using this approach, it was previously shown that the structural and functional mimicry of the idiotypic network can be exploited to elicit antibodies imparted with catalytic properties (Avalle et al., 1998; Li et al., 2012). In the first step, an antibody is raised that recognizes the active site of the antigen-enzyme. The binding site of this first antibody (Ab1) is unique with structural features complementary to those of the enzyme. This monoclonal Ab1 is selected based on its ability to inhibit the enzymatic reaction. In the second step, Ab1 -seen as a non-self protein by a distinct strain mouse's immune system- is used as immunogen to produce anti-idiotypic antibodies. Among these antibodies (Ab2), some may represent internal images of the enzyme active site.

**Figure 1:**
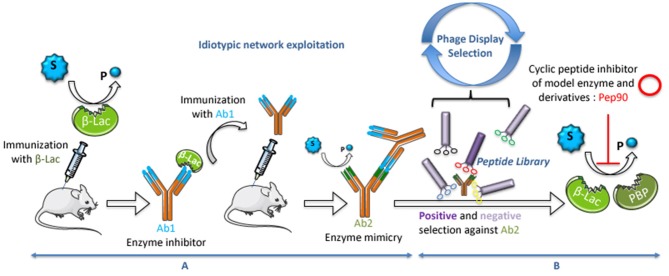
Process used to select abzymes and regulatory peptides. **(A)** Exploitation of the idiotypic network for the genesis of catalytic antibodies. **(B)** Exploitation of Phage-Display technology for the selection of inhibitory peptides.

As an example, this approach was used in the 1990s to generate an abzyme with a model enzyme (Figure 1A) (Avalle et al., 1998). This was performed by immunizing mice with the ß-lactamase from *Bacillus cereus*, preparing monoclonal hybridoma cell lines and screening for antibodies raised against the active site of the enzyme. One hundred fifty monoclonal antibodies specific for the enzyme were obtained. One of them, 7AF9, was selected as a good candidate for its inhibitory effect of the enzyme. Mice were immunized with 7AF9 and Ab2 hybridoma clones were tested for their affinity to Ab1 together with their ability to hydrolyze substrates of the enzyme. The Ab2 9G4H9 was selected as a good catalyst for both penicillinic substrates (ampicillin, k_cat_ = 2.1 min^−1^) and cephalosporins (PADAC, k_cat_ = 2.3 × 10^−3^ min^−1^). 9G4H9 is considered as a functional mimic of ß-lactamase, with a lower catalytic efficiency. To investigate the structural mimicry of 9G4H9, its variable regions were sequenced. Interestingly, the sequences did not display any significant homology to the primary structure of the model enzyme. However, three-dimensional structural modeling of the 9G4H9 variable regions of the light chain revealed some structural homology with both the active site of ß-lactamase and the penicillin-binding protein (PBP), two enzymes that have evolved from a common ancestor (Goffin and Ghuysen, 1998). The 3D superposition of the three active sites suggests the presence of most of the catalytic residues in Ab2, in a close orientation of that present in ß-lactamase and PBP (Padiolleau-Lefèvre et al., 2003). Site-directed mutagenesis of these catalytic residues confirmed their implication in ß-lactam ring hydrolysis (Phichith et al., 2009). As expected, the anti-idiotypic pathway exploited to select 9G4H9 has provoked a dramatic decrease in the catalytic efficiency when compared with the initiating catalyst, but 9G4H9 could be considered as a catalyst endowed with unique catalytic properties, intermediate between ß-lactamase and PBP, with structural features related to the two proteins. In addition, it has to be underlined that the original abzyme catalytic activity can be improved. Indeed, Golovin et al. (2017) showed the possibility of applying *in silico* approaches, such as quantum mechanics/molecular mechanics calculations, to determine the abzyme mutants with an optimal catalytic activity.

From these observations it can be proposed that both structural and functional properties can be transferred from one protein, the enzyme, to another protein, the Ab2 9G4H9, via the imprinting of the active site by an Ab1. However, it must be noted that if the structures and functions are closely related, the primary structures of the enzyme and its related catalytic anti-idiotypic antibody totally differ.

## Peptides as structural biological imprints of protein targets

On the other hand, an increasing interest concerns peptides as source of potential new therapeutic agents (Dadar et al., 2018), together with their use to complement existing genomic methodologies for deciphering complex biological processes (Delvaeye et al., 2018). Peptides are particularly well-suited to be modulators of protein functions. It is however often argued that the utility of peptides as bioactive protein mimics is limited both by their high sensitivity to proteases and the high flexibility of linear peptides which hampers the mimicry of conformationally defined less flexible protein structures. These apparent limitations can however be often overcome by conformationally constrained peptides through cyclization, or by incorporation of chemical moieties into the peptide sequence. Another benefit to cyclic peptides is their enhanced protein binding affinity.

**Figure 2:**
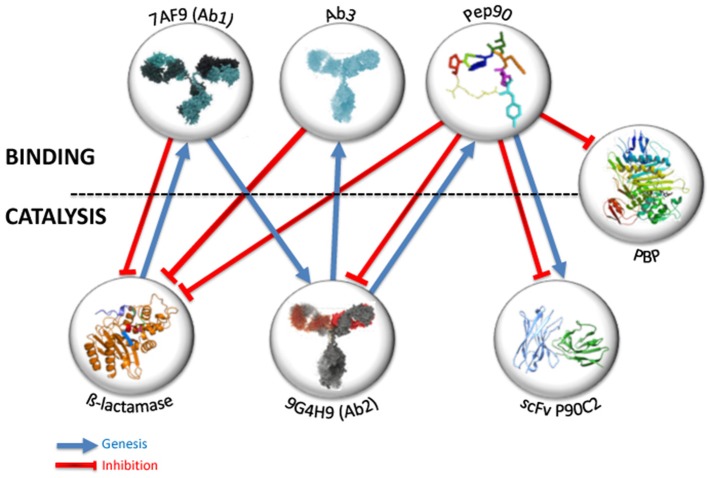
Key figure: structural and functional information transfer through rational selections. Blue arrows indicate that genesis or selection of target lies on initial entities. Red lines illustrate inhibition effect of entities on catalysts.

To investigate the possible transfer of structural information between proteins and cyclic peptides, the catalytic 9G4H9 was chosen as model. A random library displaying 4.5 × 10^9^ cyclic heptapeptides on the surface of bacteriophages was used in a two-step selection procedure. The first selection step aimed at enriching the library in species binding to the whole Ab2 molecule (Figure 1B), the second step was to eliminate peptides binding to part of the molecule other than the catalytic site (Yribarren et al., 2003). Among the four peptides selected after this second selection step, one of them (Pep90) was found to bind with a high affinity to 9G4H9 (K_D_ = 74 nM). Pep90 was also found to competitively inhibit the ß-lactamase activity of 9G4H9 and to compete with BLIP, a naturally occurring peptide previously described to inhibit the ß-lactamase enzyme (Phichith et al., 2010), for binding to 9G4H9 active site. On the other hand, site-directed mutagenesis of both Pep90 and 9G4H9 key residues has clearly demonstrated the specificity of Pep90 binding to the catalytic site of the antibody. A surprising finding arrived when Pep90 was assayed for binding and inhibition of ß-lactamases and PBP from different classes and from different bacterial strains (Figure 2).

## Peptides as targets for the selection of bioactive proteins

From all these observations, it seems that mimicry between proteins and/or peptides allows the selection of molecular entities that partially represent a structural and/or functional image of the original molecule. This generally results in the selection of catalysts (abzymes) with a lower functional efficiency and, for inhibitory peptides, in molecules with a broader specificity when compared with classical chemical inhibitors. However, very few studies have concerned the characterization of proteins that could be selected by using peptides mimicking protein active sites as templates.

If we follow the model systems described above as an example, the question is what could be the binding and eventually catalytic properties of antibodies selected against Pep90. To try to answer this question, a phage-displayed scFv library was constructed (Shahsavarian et al., 2014). This latter represents the immune repertoire of murine models of different genetic background and immune status, leading to a scFv library of size 2.7 × 10^9^. Seven rounds of panning of the pooled library were performed against Pep90. Three different scFv were obtained, characterized for binding to the cyclic peptide. Surprisingly, the three selected scFv displayed on phage surface exhibit a ß-lactamase activity for hydrolysis of nitrocefin and fluorocillin, with a higher catalytic activity for one of them. The three scFv have different primary structure. Nevertheless, a structural modeling suggests that all contains potential catalytic residues that could form a catalytic triad (Shahsavarian et al., 2017). So, the use of a small constraining peptide, with only 7 amino acids, as a template for the selection of phage-display antibody fragments, has resulted in the isolation of three scFv fragments with a ß-lactamase activity. Pep90, selected against Ab2 9G4H9 obtained by mimicry of ß-lactamase is not only able to inhibit 9G4H9, ß-lactamase enzymes and penicillin binding proteins, but it contains in its structure and in its amino acid composition an information sufficient to preferentially select antibodies with ß-lactamase activity within a murine phage-display library. Finally, thanks to the manipulation of the idiotypic network and appropriate selection against protein or peptide targets, we have come full circle.

## Possible physiological implications of the mimicry properties

The idiotypic network is involved in the physiological regulation known as idiotypic suppression. This also induces mimicry implementation with associated physiopathological consequences.

All the experimental observations described above led us to conclude that the immune system, with the well-known properties of the idiotypic network, as well as very small biomolecular structures like cyclic peptides, can transfer not only a structural, but also a functional information from a protein to another one (Figure 2). The protein/protein mimicry obtained for the biological function of selected proteins allows not only the specific binding of an entity to another one, but even a more sophisticated mechanism like catalysis. These observed properties could have important implications if we consider their possible occurrence at the physiological level. We have already suggested, according to Pierre Grabar (1975), that antibodies, and especially abzymes, may not only be involved in immune reaction against pathogens, but could interact with metabolism according to the metabolic need (Friboulet et al., 1999). The antibodies involved in catalytic reactions could result from a particular context implying abnormal concentration of molecules acting as transition state analogs and leading -by successive mimicry process- to such catalysts, as exemplified by our results showing that a cyclic heptapeptide induces the selection of antibodies endowed with a catalytic activity. They also could pre-exist at a low level in a specific physiopathological context, and thus explain the link frequently mentioned between autoimmunity and abzymes (Belogurov et al., 2009). This plausible link between autoimmunity and abzymes, alternatively beneficial or deleterious for the health, remains one of the most interesting outstanding questions regarding natural abzymes described in the literature. Finally, the question whether the catalytic function of antibodies is encoded by the germline repertoire or acquired due to somatic mutations has already been evoked (Le Minoux et al., 2012) but must still be investigated.

Molecular mimicry is now firmly considered as the basis of many autoimmune disorders where a foreign antigen shares sequence or structure similarities with self-antigens (Cohen, 2001; Chastain and Miller, 2012; Cusick et al., 2012; Pahari et al., 2017). As an example, in multiple sclerosis, it was proposed that an antigen-specific T-cell receptor cross-react with a self-antigen, the myelin-basic-protein (MBP) and a peptide analogous to part of a viral antigen, the polymerase of the Epstein-Barr virus (Lang et al., 2002). It was later shown that antibodies induced against Epstein-Barr latent membrane protein 1 during virus infection cross-react with MBP, and might act as inflammatory trigger by reacting with MBP (Gabibov et al., 2011). Finally, mimicry properties should be regarded as source for potential applications, as exemplified by the exploitation of peptidomimetics to produce vaccines. Many peptide vaccines are under development, such as vaccine for virus, bacteria, but also as therapeutic anti-cancer vaccines for pancreatic cancer, melanoma, non-small cell lung cancer, and others (Li et al., 2014; Bezu et al., 2018).

## Conclusion

In this paper, we discuss an important work aiming the selection of catalysts or regulator proteins, either through idiotypic network, illustrating a “mise en ab(z)yme,” or thanks to phage-display technology. In retrospect, analysis of data shows as expected that not only is the function brought by a structural information rather than a sequential one, but more surprisingly also that such a structural information copy can occur spontaneously and lead to a pathological situation. It will be essential to further investigate this ability of biological systems in order to understand -and above all exploit it- to propose bioinspired molecules with diagnostic or therapeutic values.

## Author Contributions

BA and SP-L performed experimental conception, analysis, and interpretation of data. AF designed the study. All authors contributed to manuscript writing and approved the submitted version.

### Conflict of Interest

The authors declare that the research was conducted in the absence of any commercial or financial relationships that could be construed as a potential conflict of interest.
